# Factors Moderating the Link between Personal Recounts of COVID-19 Vaccine Side Effects Viewed on Social Media and Viewer Postvaccination Experience

**DOI:** 10.3390/vaccines10101611

**Published:** 2022-09-26

**Authors:** Winston Tan, Ben Colagiuri, Kirsten Barnes

**Affiliations:** School of Psychology, Faculty of Science, University of Sydney, Camperdown, NSW 2006, Australia

**Keywords:** social learning, vaccine, side effects, COVID-19, social media

## Abstract

While social media exposure is known to influence vaccine hesitancy, its impact on postvaccination experience remains relatively unknown. This retrospective cross-sectional study explored whether various psychosocial and individual factors moderate the association between social media exposure to personal recounts of COVID-19 vaccine side effects and the viewer’s subsequent postvaccination side effect experience. Adults residing in Australia, who were fully vaccinated with two COVID-19 vaccine doses (*n* = 280) completed an online survey. The more severe the personal recounts of post-COVID-19 vaccination side effects participants were exposed to on social media, the more severe their own postvaccination side effects were following both their first *(β* = 0.261, *p* < 0.001) and second dose (*β* = 0.299, *p* < 0.001). This association was stronger among those with greater vaccine side effect worry, elevated negative emotional states such as anxiety and stress, and a stronger proclivity for using social media over mainstream media for COVID-19 vaccine side effect information. As such, not only does social influence appear to exacerbate or trigger postvaccination side effects, but a range of psychosocial and situational factors moderate this association. Health organisations and government bodies could minimise the negative effects of social media exposure in future health outbreaks by countering treatment misperceptions on social media platforms as they arise.

## 1. Introduction

As the global community continues to battle with the COVID-19 pandemic, adverse events following COVID-19 vaccination has lingered as a significant talking point. This is particularly troubling considering the substantial diffusion of negative vaccine-related information occurring through unregulated channels such as social media [1]. Most research in this area has focused on the role of antivaccine content and perceptions of media in fuelling vaccine hesitancy (e.g., [2,3,4]). However, while social media exposure may reduce vaccine intent, whether it can augment the experience of side effects postvaccination remains relatively untested, with nothing known about the characteristics of those most at risk.

Although vaccine side effects are common as part of the body’s innate immune response [5], psychosocial factors, such as worry about COVID-19, expectations for side effects, and depressive symptoms have been associated with COVID-19 vaccine reactogenicity [6]. Further, a recent systematic review of COVID-19 vaccine randomised clinical trials found that up to 76% of systemic adverse events could be attributed to a nocebo response [7], suggesting that negative preconceptions regarding COVID-19 vaccine side effects may exacerbate their experience [8]. Current evidence therefore demonstrates that a range of factors, including those that may be acquired through social media exposure, such as worry, are sufficient to exacerbate or trigger COVID-19 vaccine side effects.

Unfortunately, vaccine hesitancy has been highly prevalent since the beginning of the global vaccination rollout, particularly across high-income countries, such as Australia [9]. One significant contributor to this hesitancy includes worry surrounding side effects [10], with many individuals indicating that they wanted to observe how the vaccine affected others before receiving it themselves [11]. This type of social information can be readily amassed from sources such as social media and similar online platforms. While many turned to social media throughout the pandemic to stay connected with friends and family and circulate medical information within their closed networks [12], these platforms also aggregated individuals into a global public network where there exists significant potential for exposure to social information posted by other users, including details of adverse events experienced.

However, a paucity of research exists concerning how exposure to this social information on social media may influence the viewer’s postvaccination experience of side effects, with only one study having explored the association. In this previous study [13], as exposure to social media posts concerning COVID-19 vaccine side effects increased in frequency and severity, so did the severity of postvaccination side effects subsequently experienced by the viewer. This relationship was mediated by negative expectations for side effects, a mechanism known to underlie nocebo effects. As reviewed, while other psychosocial and nocebo-related contextual factors predict an individual’s postvaccination experience [6], whether they moderate the effect of social media exposure on COVID-19 vaccine side effects is unknown. Similarly, it is unclear whether perceptions of social media (e.g., preference and credibility) strengthen any association between social media exposure and COVID-19 vaccine side effects.

To bridge this gap, the present study first endeavoured to substantiate that social media exposure was related to the experience of COVID-19 vaccine side effects. It was thus hypothesised that the severity of side effects seen reported by other users on social media would be associated with subsequent side effect severity experienced. Importantly, several moderating factors were tested. These included the psychosocial variables found to predict COVID-19 vaccine reactogenicity [6] but extended this research to investigate the belief in common COVID-19 vaccine misconceptions as well as perceptions of different media sources. As such, the current study not only provides evidence regarding the characteristics of individuals at risk of developing side effects after social media exposure, but also the type of beliefs and perceptions that potential interventions should target to reduce such an association.

## 2. Materials and Methods

The present retrospective, cross-sectional study was conducted with 285 participants who completed a single online survey on the survey platform Qualtrics. Responses from participants with more than one entry (*n* = 4) and a response completed faster than the preregistered exclusion criteria of under 8 min (*n* = 1) were removed, leaving a final sample of 280 (see Table 1 for demographic characteristics). Initial contact and recruitment took place via advertisements on social media platforms Facebook, Instagram, and Reddit from August 2021 to January 2022. Participants resided in Australia and were fully vaccinated with a two-dose vaccine (AstraZeneca, Pfizer, Moderna). Participants were required to be fluent in English and use social media daily for nonwork reasons. Participants were informed of the survey length, data storage, and investigator details via an information statement. Digital consent was subsequently obtained and a debrief statement was provided upon conclusion of the study. Ethical approval was granted by the University of Sydney Human Research Ethics Committee (protocol 2021/492).

The survey included three key variables: (1) severity of side effects seen reported by other social media users prior to vaccination, (2) the participants’ side effect experience after their first dose, and (3) after their second dose. Each was measured via a modified version of the Generic Assessment of Side Effects questionnaire (GASE) [14]. All three versions included 21 sum-scored items: 18 relating to COVID-19 vaccine side effects (e.g., headache and fatigue) and 3 open-response items concerning side effects not otherwise listed. Regarding social media reports, participants indicated whether they had seen each of the 21 side effects listed in the GASE reported by others on social media. Scores ranged from 0 (reports regarding the specific side effect not seen) to 10 (severe reports regarding the specific side effect seen). In the personal experience versions, participants indicated whether they had personally experienced the 21 side effects listed in the GASE from 0 (specific side effect not experienced) to 10 (severe experience of the specific side effect). Participants also completed self-report measures related to their emotional states and other COVID-19-vaccine-related perceptions they had at the time of receiving the vaccine (see Table S1 for brief explanations of variables). Moderation analyses were conducted using Model 1 of PROCESS macro 4.0 [15] in SPSS Version 24.0 (IBM Corp, Armonk, NY, USA). Bootstrapping with 10,000 samples was conducted to determine 95% confidence intervals (CIs) that determined statistical significance. All other analyses were performed using SPSS Version 24 with an alpha of 0.05.

## 3. Results

Two simple linear regressions were run to identify whether the severity of the side effects seen reported by other social media users (exposure) predicted the severity of the side effects experienced by participants in the three days after they received their first (experience 1) and second (experience 2) vaccine dose. Both models explained a significant proportion of variance in postvaccination side effects (experience 1: *F*(1,278) = 20.392, *p* < 0.001, *R*^2^ = 0.07; experience 2: *F*(1,278) = 27.264, *p* < 0.001, *R*^2^ = 0.09), with exposure being a significant predictor (experience 1: *β* = 0.261, *p* < 0.001/experience 2: *β* = 0.299, *p* < 0.001).

Secondary variables (as described in Table S1) were subsequently explored as potential moderators of the social media exposure–first dose experience association. Analyses (see Table 2) revealed several significant moderators. Regarding media platforms, a preference for COVID-19 vaccine side effect information from social media over mainstream media (*b* = 0.37, *t*(276) = 4.92, *p* < 0.001), the strength of this preference (*b* < 0.01, *t*(276) = 4.93, *p* < 0.001), a stronger belief in social media as a credible source of COVID-19 vaccine side effect information (*b* < 0.01, *t*(276) = 3.83, *p* < 0.001), and a weaker belief in mainstream media as a credible source (*b <* 0.01, *t*(276) = −2.27, *p* = 0.024) strengthened the association between social media exposure and postvaccination experience. Greater COVID-19 vaccine worry (*b* < 0.01, *t*(276) = 4.63, *p* < 0.001), stronger beliefs in common COVID-19 vaccine misconceptions, elevated depressive, anxiety and stress-related symptoms *(b* = 0.22, *t*(276) = 5.03, *p* < 0.001), and more severe side effects experienced by individuals personally known to the participant (*b* = 0.03, *t*(234) = 2.68, *p* = 0.008) also increased the magnitude of the association between social media exposure and first-dose experience. Comparable outcomes were additionally found for the social media exposure–second dose experience association (see Table 3).

## 4. Discussion

The present study demonstrates that exposure to personal recounts of COVID-19 vaccine side effects on social media is predictive of one’s own postvaccination experience and that several factors moderate this association, including psychosocial influences, social media perceptions, and COVID-19 vaccine misperceptions and worry. These findings therefore expand on previous research [13], highlighting not only how vaccine-related social information obtained from social media platforms is related to our own health outcomes, but also identifying those who are potentially at greater risk. For instance, the association between social media exposure and side effect experience was stronger when examined in the context of nocebo-related psychosocial factors. This extends previous research, which found general depressive symptoms were a significant predictor of side effect experience [6], to demonstrate that those individuals who also score highly on the dispositional factors of anxiety and stress also appear at increased risk of side effect exacerbation after social media exposure. Importantly, the results were not limited to these general psychosocial predictors. For example, a greater worry about COVID-19 vaccine side effects strengthened the exposure–experience association across both doses.

Further, we demonstrated for the first time that a greater preference for, and perceived credibility of, social media as a COVID-19 vaccine side effect information source intensified the predictive effect of social media exposure. Conversely, the effect was strengthened when mainstream media was perceived as a noncredible source, while a previous study [13] found that asking participants whether mainstream media (i.e., news stories) gave the impression that vaccines caused side effects did not predict the severity of their own experience. The current research thus demonstrates that, rather than general impressions, it is the interpretation of those impressions (i.e., their credibility as an information source) that matters. While the presence of side effect information cannot be suppressed from social media, potential interventions can strive to alter one’s perception of its credibility, a contextual factor known to alter the evaluation of information truthfulness [16]. This may also serve to shift beliefs in common COVID-19 vaccine misconceptions, an additional moderator observed here. Given that a greater severity of side effects experienced by individuals personally known to the participant was also a significant moderator, the present results demonstrate that information from closed social networks, as well as more distant social others can have a considerable effect on our health outcomes and a multitude of psychosocial factors can bolster this association. Government bodies and health organisations may endeavour to utilise social media more substantially upon future disease outbreaks to prevent potential damage caused by social media exposure. One possibility would be to highlight how negative attitudes resulting from social information may influence vaccine perceptions and experiences, as has been effective in minimising side effects to other treatments and outcomes (e.g., [17,18]).

There are various strengths to this study. The use of a community sample recruited through social media platforms increased the likelihood that those most likely to be exposed to health-related social information on social media were represented. Moreover, this study included second-dose data, demonstrating that the effect of social media exposure and its interaction with the tested moderators on side effect experience appeared persistent over time. However, one limitation of the present study is that retrospective data may have been subject to recall bias, meaning measures were more reliant on semantic memory rather than the participants’ affective and physical states at the time of vaccination. Relatedly, due to the cross-sectional nature of these data, it is possible that responses relating to the first-dose experience were influenced by the recall of the second-dose experience. Greater specificity when measuring side effects associated with each vaccine type may also be advantageous. Individuals could have been exposed to reports of side effects limited to a vaccine that they did not subsequently receive, which may have reduced the effect size observed in the present analysis. Moreover, relative to previous research in this area (e.g., *n* = 551 as in [6,13]), the sample size of this study was relatively small. While we detected an effect of medium magnitude for the primary outcome, future research may wish to replicate these outcomes with larger sample sizes. Future studies may also find it informative to record the social media platforms participants use, as these platforms may differ in the social information they carry (e.g., users who feel more strongly about sharing their negative experiences may congregate on certain platforms).

## 5. Conclusions

Nevertheless, this study provided valuable insights by highlighting that a multitude of factors were involved in the link between exposure to details of adverse events posted by others on social media and its potential influence over an observer’s own subsequent experience. Given that side effect information presented on social media is likely to have the most detrimental effect among those who prefer this source of social information and find it more credible, government bodies and health organisations may benefit from increasing their presence on social media platforms to minimise future adverse events. In summary, while the potential dangers of social media exposure on vaccine hesitancy are remarkably apparent, the present research highlights how social media may have a further reach, even negatively affecting those willing to be vaccinated. Interventions tailored to reduce the burden of social media exposure, particularly in times of high stress or anxiety, will therefore provide pathways through which vaccine acceptance and adherence can be increased.

## Figures and Tables

**Table 1:** Demographic Characteristics.

Variable	Mean (SD)/*n* (%)
Age (mean)	41.74 (16.05)
Age ranges	
18–29	78 (27.9%)
30–39	67 (23.9%)
40–49	31 (11.1%)
50–59	52 (18.6%)
>60	45 (16.1%)
Gender	
Male	95 (33.9%)
Female	173 (61.8%)
Other	9 (3.2%)
Preferred not to say	3 (1.1%)
Highest educational attainment	
Less than high school	3 (1.1%)
High school	29 (10.4%)
Some college but no degree	27 (9.6%)
Graduate diploma or certificate	65 (23.2%)
Bachelor’s degree	112 (40.0%)
Master’s degree	29 (10.4%)
Doctoral degree	9 (3.2%)
Professional degree (JD, MD)	6 (2.1%)
Time since first dose received (at time of taking survey)	
Less than a month ago	8 (2.9%)
1–2 months ago	51 (18.2%)
2–3 months ago	73 (26.1%)
3–4 months ago	55 (19.6%)
4–5 months ago	45 (16.1%)
Over 6 months ago	48 (17.1%)
Political ideology	
Left	147 (52.5%)
Centre	37 (13.2%)
Right	55 (19.6%)
Preferred not to say	41 (14.6%)
First-dose vaccine	
Pfizer	169 (60.4%)
AstraZeneca	101 (36.1%)
Moderna	10 (3.6%)
Second-dose vaccine	
Pfizer	168 (60.0%)
AstraZeneca	102 (36.4%)
Moderna	10 (3.6%)

**Table 2:** First dose moderation analyses—bold variables are significant moderators (note: each moderator was investigated in separate moderation models).

	Model Variable								
	Social Media Exposure on First-Dose Side Effects			Moderator Variable (As Stated in Column 1) on First-Dose Side Effects			Social Media × Moderator Interaction on First-Dose Side Effects		
Moderator	B Coeff (SE)	*t* (*p*-Val)	95% CI [LL, UL]	B Coeff (SE)	*t* (*p*-Val)	95% CI [LL, UL]	B Coeff (SE)	*t* (*p*-Val)	95% CI [LL, UL]
1. **Social Media** **Preference**	−0.33 (0.01)	−3.23 (**0.001**)	[−0.53, −0.13]	9.03 (3.28)	2.75 (**0.006**)	[2.56, 15.48]	0.37 (0.08)	4.92 (**<0.001**)	[0.22, 0.52]
2. **Preference** **Strength**	0.12 (0.03)	3.66 (**<0.001**)	[0.06, 0.19]	0.17 (0.05)	3.45 (**<0.001**)	[0.07, 0.27]	<0.01 (<0.01)	4.93 (**<0.001**)	[<0.01, 0.01]
3. Preference (Statistics and Figures)	0.16 (0.03)	4.55 (**<0.001**)	[0.09, 0.23]	−0.06 (0.03)	−1.80 (0.073)	[−0.12, <0.01]	<−0.01 (<0.01)	−1.44 (0.150)	[<−0.01, <0.01]
4. Preference (Original Post)	0.16 (0.04)	4.44 (**<0.001**)	[0.09, 0.23]	0.02 (0.04)	0.50 (0.617)	[−0.06, 0.10]	<−0.01 (<0.01)	0.77 (0.939)	[<−0.01, <0.01]
5. **Mainstream** **Media Credibility**	0.11 (0.04)	3.10 (**0.002**)	[0.04, 0.18]	−0.18 (0.05)	−3.53 (**<0.001**)	[−0.28, −0.08]	<−0.01 (<0.01)	−2.28 (**0.024**)	[<−0.01, <−0.01]
6. **Social Media Credibility**	0.14 (0.03)	4.35 (**<0.001**)	[0.08, 0.21]	0.21 (0.06)	3.48 (**<.001**)	[0.09, 0.32]	<0.01 (<0.01)	3.83 (**<0.001**)	[<0.01, <0.01]
7. COVID-19 Worry	0.16 (0.04)	4.65 (**<0.001**)	[0.09, 0.23]	−0.05 (0.05)	−1.00 (0.318)	[−0.14, 0.05]	<−0.01 (<0.01)	−1.39 (0.163)	[<−0.01, <0.01]
8. **COVID-19** **Vaccine Worry**	0.10 (0.03)	3.13 (**0.002**)	[0.04, 0.17]	0.20 (0.04)	4.60 (**<0.001**)	[0.11, 0.28]	<0.01 (<0.01)	4.63 (**<0.001**)	[<0.01, <0.01]
9. eHealth Literacy	0.16 (0.04)	4.66 (**<0.001**)	[0.10, 0.23]	−0.27 (0.27)	−1.01 (0.311)	[−0.80, 0.26]	<−0.01 (<0.01)	−1.11 (0.268)	[−0.02, 0.01]
10. Intolerance of Uncertainty	0.18 (0.04)	4.97 (**<0.001**)	[0.11, 0.25]	0.40 (0.14)	2.84 (**0.005**)	[0.12, 0.67]	<0.01 (<0.01)	1.39 (0.163)	[<−0.01, 0.01]
11. **Depression**, **Anxiety, and Stress Score**	0.12 (0.03)	3.58 (**<0.001**)	[0.05, 0.18]	12.42 (2.37)	5.24 (**<0.001**)	[7.75, 17.10]	0.22 (0.04)	5.03 (**<0.001**)	[0.13, 0.30]
12. **Experience of Known Others**	0.04 (0.04)	1.02 (0.307)	[−0.04, 0.12]	5.70 (0.82)	6.97 (**<0.001**)	[4.10, 7.32]	0.04 (0.01)	2.68 (**0.008**)	[0.01, 0.06]
13. **Vaccine Belief Misconceptions**	0.08 (0.03)	2.47 (**0.014**)	[0.02, 0.14]	0.06 (0.01)	6.34 (**<0.001**)	[0.04, 0.09]	<0.01 (<0.01)	4.76 (**<0.001**)	[<0.01, <0.01]

The symbol × in the fourth column ‘Social Media × Moderator Interaction On Second-Dose Side Effects’ represents the calculation of the interaction term between social media exposure and the secondary variable.

**Table 3:** Second dose moderation analyses—bold variables are significant moderators (note: each moderator was investigated in separate moderation models).

	Model Variable								
	Social Media Exposure on Second-Dose Side Effects			Moderator Variable (As Stated in Column 1) on Second-Dose Side Effects			Social Media × Moderator Interaction on Second-Dose Side Effects		
Moderator	B Coeff (SE)	*t* (*p*-Val)	95% CI [LL, UL]	B Coeff (SE)	*t* (*p*-Val)	95% CI [LL, UL]	B Coeff (SE)	*t* (*p*-Val)	95% CI [LL, UL]
1. **Social Media** **Preference**	−0.26 (0.10)	−2.50 (**0.013**)	[−0.46, −0.06]	12.55 (3.36)	3.74 (**<0.001**)	[5.94, 19.17]	0.34 (0.07)	4.36 (**<0.001**)	[0.18, 0.49]
2. **Preference Strength**	0.14 (0.03)	4.24 (**<0.001**)	[0.07, 0.21]	0.23 (0.05)	4.55 (**<0.001**)	[0.13, 0.33]	<0.01 (<0.01)	4.50 (**<0.001**)	[<0.01, 0.01]
3. Preference (Statistics and Figures)	0.19 (0.04)	5.27 (**<0.001**)	[0.12, 0.26]	−0.04 (0.03)	−1.27 (0.206)	[−0.10, 0.02]	<−0.01 (<0.01)	−1.63 (0.105)	[<−0.01, <0.01]
4. Preference (Original Post)	0.18 (0.04)	4.88 (**<0.001**)	[0.11, 0.25]	<0.01 (0.04)	0.34 (0.731)	[−0.07, 0.10]	<−0.01 (<0.01)	−1.18 (0.238)	[<−0.01, <0.01]
5. **Mainstream** **Media Credibility**	0.14 (0.04)	3.67 (**<0.001**)	[0.06, 0.21]	−0.19 (0.05)	−3.52 (**<0.001**)	[−0.29, −0.08]	<−0.01 (<0.01)	−2.73 (**0.007**)	[<−0.01, <−0.01]
6. **Social Media Credibility**	0.17 (0.03)	5.09 (**<0.001**)	[0.11, 0.24]	0.23 (0.06)	3.78 (**<0.001**)	[0.11, 0.35]	<0.01 (<0.01)	3.74 (**<0.001)**	[<0.01, <0.01]
7. COVID-19 Worry	0.19 (0.04)	5.43 (**<0.001**)	[0.12, 0.26]	−0.08 (0.05)	−1.70 (0.090)	[−0.18, 0.01]	<−0.01 (<0.01)	−1.65 (0.099)	[<−0.01, <0.01]
8. **COVID-19** **Vaccine Worry**	0.13 (0.03)	3.85 (**<0.001**)	[0.06, 0.20]	0.22 (0.04)	4.99 (**<0.001**)	[0.13, 0.31]	<0.01 (<0.01)	4.14 (**<0.001**)	[<0.01, <0.01]
9. eHealth Literacy	0.19 (0.04)	5.38 (**<0.001**)	[0.12, 0.27]	−0.44 (0.27)	−1.61 (0.108)	[−0.98, 0.01]	<−0.01 (<0.01)	−1.02 (0.308)	[<−0.01, <0.01]
10. Intolerance of Uncertainty	0.18 (0.03)	5.24 (**<0.001**)	[0.11, 0.25]	0.32 (0.14)	2.33 (**0.021**)	[0.05, 0.58]	<−0.01 (<0.01)	−0.48 (0.629)	[<−0.01, 0.01]
11. **Depression,** **Anxiety, and Stress Score**	0.15 (0.03)	4.42 (**<0.001**)	[0.08, 0.22]	11.00 (2.52)	4.36 (**<0.001**)	[6.04, 15.98]	0.18 (0.05)	3.80 (**<0.001**)	[0.08, 0.27]
12. **Experience of Known Others**	0.08 (0.04)	2.00 (**0.047**)	[<0.01, 0.16]	4.16 (0.87)	4.77 (**<0.001**)	[2.44, 5.88]	0.06 (0.01)	3.93 (**<0.001**)	[0.03, 0.09]
13. **Vaccine Belief Misconceptions**	0.11 (0.03)	3.40 (**0.001**)	[0.05, 0.18]	0.06 (0.01)	5.87 (**<0.001**)	[0.04, 0.08]	<0.01 (<0.01)	3.90 (**<0.001**)	[<0.01, <0.01]

The symbol × in the fourth column ‘Social Media × Moderator Interaction On Second-Dose Side Effects’ represents the calculation of the interaction term between social media exposure and the secondary variable.

## Data Availability

The data presented and a version of the questionnaire used in this study are openly available in Open Science Framework at https://osf.io/tp6cs/ (accessed on 21 August 2022).

## Supplementary Materials

The following supporting information can be downloaded at: https://www.mdpi.com/article/10.3390/vaccines10101611/s1, Table S1: Secondary Variable Descriptions.
