# Supplementary material for: Factors Moderating the Link between Personal Recounts of COVID-19 Vaccine Side Effects Viewed on Social Media and Viewer Postvaccination Experience
Source: Vaccines (Basel). 2022 Sep 26;10(10):1611. doi: 10.3390/vaccines10101611 (PMC9610806; doi:10.3390/vaccines10101611)
Supplement: Supplementary file 1 [file vaccines-10-01611-s001.zip › vaccines-1902376-supplementary.pdf]

Supplementary Material

Table S1. Secondary Variable Descriptions.

Variable	Item	Scoring	Interpretation
Social Media Preference	Participants indicated whether they preferred mainstream or social media when seeking information related to the experience of COVID-19 vaccine side effects between 1 – Mainstream media and 2 – Social media (forced choice)		Higher score = preference for social media
Preference Strength	Participants indicated their strength of preference for mainstream or social media from −50 (Mainstream media) to 50 (Social media)		Higher score = greater preference for social media
Preference for Statistics and Figures over Personal Stories (A)	Participants rated how valuable they found the following pieces of information when reading social media posts about COVID-19 vaccine side effects from 0 (Not at all valuable) – 100 (Extremely valuable):<br><br>1) Statistics and figures posted by the original poster<br>2) Statistics and figures presented by other posters in the comments or replies<br>3) Personal stories posted by the original poster<br>4) Personal stories posted by other posters in the comments or replies	A = (1 + 2) - (3 + 4)	Higher score = greater preference for statistics and figures over personal stories
Preference for Original Posts over comments or replies (B)		B = (1 + 3) - (2 + 4)	Higher score = greater preference for original posts over comments or replies
Mainstream Media Credibility	Participants rated how credible they believed mainstream media to be in providing accurate information related to the experience of COVID-19 vaccine side effects from 0 (Not at all credible) – 100 (Very credible)		Higher score = greater belief in mainstream media as a credible source
Social Media Credibility	Participants rated how credible they believed social media to be in providing accurate information related to the experience of COVID-19 vaccine side effects from 0 (Not at all credible) – 100 (Very credible)		Higher score = greater belief in social media as a credible source
COVID-19 Worry	Measured using the COVID-19 worry scale (Ahmed et al., 2020)	Mean average of all items	Higher score = greater worry about COVID-19
COVID-19 Vaccine Worry	Participants indicated how worried they were about experiencing side effects from the COVID-19 vaccine from 0 (Not at all worried) – 100 (Extremely worried)		Higher score = greater worry about experiencing side effects
eHealth Literacy	eHealth Literacy Scale (Norman & Skinner, 2006)	Sum of all items	Higher score = greater eHealth literacy
Intolerance of Uncertainty	Intolerance of Uncertainty Scale – Short Form (Carleton et al., 2008)	Sum of all items	Higher score = greater intolerance of uncertainty
Depression, Anxiety, & Stress Score	Depression, Anxiety, and Stress Scale (Osman, 2012)	Mean average of the three subscales	Higher score = greater overall depression, anxiety,

			& stress-related symptoms
Experience of Known Others	Participants indicated how severe the COVID-19 vaccine side effects experienced by those personally known to them were from 0 (No side effects) – 10 (Severe side effects)		Higher score = more severe side effect experience of personally known others
Vaccine Belief Misconceptions	Participants rated the extent to which they believed in eight common vaccine misconceptions (e.g., The COVID-19 vaccines can affect a person's fertility) from −50 (Definitely untrue) – 50 (Definitely true)	Sum of all items	Higher score = greater belief in vaccine misconceptions
